# High Glucose Induced Changes in Human VEC Phenotype in a 3D Hydrogel Derived From Cell-Free Native Aortic Root

**DOI:** 10.3389/fcvm.2021.714573

**Published:** 2021-08-12

**Authors:** Sergiu Cecoltan, Letitia Ciortan, Razvan D. Macarie, Mihaela Vadana, Andreea C. Mihaila, Monica Tucureanu, Mihaela-Loredana Vlad, Ionel Droc, Mihaela Gherghiceanu, Agneta Simionescu, Dan Teodor Simionescu, Elena Butoi, Ileana Manduteanu

**Affiliations:** ^1^Biopathology and Therapy of Inflammation, Institute of Cellular Biology and Pathology “Nicolae Simionescu”, Bucharest, Romania; ^2^Cardiovascular Surgery Department, Central Military Hospital, Bucharest, Romania; ^3^Victor Babeş National Institute of Pathology, Carol Davila University of Medicine and Pharmacy, Bucharest, Romania; ^4^Clemson University, Cardiovascular Tissue Engineering in Diabetes, Clemson, SC, United States

**Keywords:** 3D-valve model, endothelin-1, endothelial to mesenchymal transition, high glucose, inflammation, valvular endothelial cells, aortic valve disease

## Abstract

**Background:** Valvular endothelial cells (VEC) have key roles in maintaining valvular integrity and homeostasis, and dysfunctional VEC are the initiators and major contributors to aortic valve disease in diabetes. Previous studies have shown that HG stimulated an inflammatory phenotype in VEC. Inflammation was shown to induce endothelial to mesenchymal transition (EndMT), a process extensively involved in many pathologies, including calcification of the aortic valve. However, the effect of HG on EndMT in VEC is not known. In addition, there is evidence that endothelin (ET) is a proinflammatory agent in early diabetes and was detected in aortic stenosis, but it is not known whether HG induces ET and endothelin receptors and whether endothelin modulates HG-dependent inflammation in VEC. This study aims to evaluate HG effects on EndMT, on endothelin and endothelin receptors induction in VEC and their role in HG induced VEC inflammation.

**Methods and Results:** We developed a new 3D model of the aortic valve consisting of a hydrogel derived from a decellularized extracellular cell matrix obtained from porcine aortic root and human valvular cells. VEC were cultured on the hydrogel surface and VIC within the hydrogel, and the resulted 3D construct was exposed to high glucose (HG) conditions. VEC from the 3D construct exposed to HG exhibited: attenuated intercellular junctions and an abundance of intermediate filaments (ultrastructural analysis), decreased expression of endothelial markers CD31 and VE–cadherin and increased expression of the mesenchymal markers α-SMA and vimentin (qPCR and immunocytochemistry), increased expression of inflammatory molecules ET-1 and its receptors ET-A and ET-B, ICAM-1, VCAM-1 (qPCR and Immunocytochemistry) and augmented adhesiveness. Blockade of ET-1 receptors, ET-A and ET-B reduced secretion of inflammatory biomarkers IL-1β and MCP-1 (ELISA assay).

**Conclusions:** This study demonstrates that HG induces EndMT in VEC and indicates endothelin as a possible target to reduce HG-induced inflammation in VEC.

## Introduction

Aortic stenosis is a degenerative disease characterized by inflammation, fibrosis and calcification leading to aortic cusps stiffness, narrowing of the aortic valve opening and stenosis. Diabetes mellitus (DM) has been reported to represent a risk factor for aortic stenosis (AS) (1) and is predictive of valve degeneration at the structural and functional level. Diabetic patients have a poor prognosis for aortic valve disease and accelerated degeneration of implanted bio-prosthetic aortic valves (2). Clinical studies indicate an association of diabetes with increased risk of developing AS (3, 4) and diabetes may be considered a predicting factor for the development of AS (5). Moreover, studies in animal models suggest that diabetes may contribute to aortic valve degeneration (6, 7). Recently, we have shown that diabetes induced early molecular and functional changes in aortic heart valve tissues in a murine model of atherosclerosis (7).

Previously, using an animal model of hyperglycemia combined with hyperlipemia (HD), it was shown that the aortic valve is one of the first territories displaying pathological modifications, leading rapidly to the development of valvular atheroma (8). In HD hamsters, the changes were mostly noticed on the aortic side of the valve (fibrosa), the surface exposed to low shear forces and high hydrostatic pressure. Valvular endothelial cells (VEC) switched to a secretory and adhesive phenotype. Similar to endothelial cells (EC) from different vascular beds, VEC response to the modified microenvironment was comparable to the response observed in atherosclerotic injury, initially by modulating the constitutive functions, the selective permeability and the biosynthetic capacity, followed by endothelial dysfunction, and ultimately by injury and apoptosis (8).

In addition, acute high glucose conditions (associated with diabetes) are pro-inflammatory for VEC, inducing enhanced adhesion of monocytes by mechanisms involving cell adhesion molecules: ICAM-1, E-selectin and CD18. Compared with aortic endothelial cells, VEC were shown to be more adhesive to monocytes, results which may explain, in part, the propensity of cardiac valves toward accelerated atherosclerosis in diabetes (9).

In a prior study, using a 3D cell culture model based on gelatin populated with human valvular cells, we demonstrated that exposure of VEC to chronic high glucose conditions induces an inflammatory phenotype by increasing the expression of various mediators and activation of protein kinase C (10, 11).

However, since pharmacological approaches for the treatment of AS are unavailable, specific molecules and mechanisms associated with VEC dysfunction in diabetes still need to be uncovered. To improve our understanding of the dynamic crosstalk between cells and their matrix microenvironment as well as the cells' response to various insults, including hyperglycemia, valvular cells need to be cultured in a physiologically relevant matrix environment that can mimic the composition of the aortic valve. For this purpose, we developed a new 3D cell culture model that mimics the composition of the aortic valve leaflet by using a hydrogel derived from a decellularized aortic root extracellular matrix. Human VEC were cultured on the hydrogel surface and VIC within the hydrogel and the resulted 3D construct was exposed to high glucose (HG) conditions. Using this 3D model, we focused our analysis on the HG impact on VEC, considering that they are first respondents to HG insult. Since in our 3D model VEC and VIC were co-cultured in a physiologically relevant matrix environment composed mainly of type I collagen (similar to aortic valve fibrosa), we hypothesize that VEC phenotypic changes induced by HG, using this model, may help to better understand VEC dysfunction in aortic valve pathology induced by diabetes.

Since in previous studies we have shown that chronic HG induced an inflammatory phenotype in VEC and inflammation was shown to promote endothelial to mesenchymal transition (EndMT) (12), in the present work we explored the potential of HG to induce this process. Therefore, after an initial characterization of the 3D model, we exposed the construct to HG and analyzed the ultrastructural modifications, and evaluated the mesenchymal and endothelial biomarkers in VEC. Moreover, since endothelin (ET-1) was found to be increased by HG in other types of EC (13) and there is evidence that it acts as an early pro-inflammatory cytokine in diabetes (14), we investigated if this molecule was induced by HG in VEC and if it is involved in VEC inflammation.

## Materials and Methods

### Protocol to Obtain the Porcine Aortic Root Derived Hydrogel

ARdH was obtained by modifying a method described in Sierad et al. (15). Aortic roots were isolated from fresh swine hearts, the aortic leaflets were removed, and the remaining tissue was cut into smaller fragments and subjected to a decellularization procedure by a detergent/nuclease method as previously described (16). Briefly, the decellularization method included a hypotonic shock using ddH_2_O 24 h at 4°C, alkaline treatment to initiate cell removal (0.1 M NaOH for 2 h), detergent treatment [0.25% SDS, 0.5% Deoxycholate (Sigma, D6750), 0.5% Triton X100, 0.2% EDTA (Sigma, E6511), 0.05 M Tris-HCl pH 7.4 incubation for 48 h] and nucleic acid removal by incubation in a 720 mU/mL deoxyribonuclease (Sigma, DN25), 720 mU/mL ribonuclease (Sigma, R5503) mixture in PBS for 2 days at 37°C. Between steps, the fragments were rinsed in ddH_2_O (3 times for 15 min) and finally rinsed in 70% EtOH overnight at room temperature then stored frozen.

The decellularized porcine aortic root pieces were frozen and subsequently lyophilized (72 h at −48°C and 0.045 mBar) and weighed. After determining the dry matrix mass, 10 mM HCl acid solution and porcine intestinal pepsin (Sigma, P7125) were added to a final concentration of 160 U/mg dry matrix, yielding a suspension with the final protein concentration of 50 mg/ml. The suspension was homogenized on a magnetic stirring plate for 4 days at room temperature to ensure enzymatic digestion. The obtained viscous gel was centrifuged for 30 min, at 3,000 × g, 4°C to remove possible undigested pieces and filtered using a 100 μm stainless steel filtering unit. The gel was stored in the refrigerator no more than 1 month before use.

**Figure:**
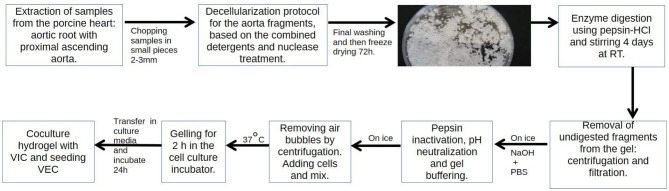
Schematic protocol for obtaining ARdH from porcine aortic roots.

### Assessment of Decellularization Efficiency by DNA Analysis

To verify decellularization efficiency, DNA was isolated from fresh aortic roots and decellularized tissue samples. Purification was performed using Wizard Genomic DNA Purification kit (Promega) and samples were quantified by reading absorbances at 260 nm on a NanoDrop 2000 (Thermo Scientific). DNA was normalized to hydrated tissue weight and expressed as ng/mg tissue.

### Evaluation of Protein Composition of ARdH by SDS-PAGE

To determine the ECM protein composition of the developed hydrogel, fresh aortic root (AR) tissue, ARdH and recombinant ECM proteins: Collagen type I (Corning, 354236), Collagen type III (Sigma, CC078), Collagen type IV (Sigma; C6745), Laminin (Sigma; L2020), and Fibronectin (Gibco; 33010018) were lysed in 2x Laemmli's electrophoresis sample buffer supplemented with 3 M urea in Tris-HCl 50 mM, pH 7.5, and incubated for 15 min at 95°C. Protein samples (30 μg protein/lane for AR and ARdH and 15 μg protein/lane for recombinant proteins) and Precision Plus Protein™ (BioRad) marker were resolved by electrophoresis on an 8% polyacrylamide gel and then stained using Coomassie Brilliant Blue G (Sigma). Imaging of protein bands was performed using a digital detection system (ImageQuant LAS 4000, Fujifilm).

### Development and Characterization of 3D Cell Culture Model of Human Aortic Valve

#### Obtaining the 3D Model With Human Valvular Cells

Primary human VEC were isolated from non-calcified cusps of human aortic valves as described in (11) and human VIC were purchased from Innoprot (cryo-preserved valvular interstitial cells P10462). VEC phenotype was validated by detection of endothelial-specific markers CD31 (PECAM-1) and Von Willebrand Factor (vWF), while VIC phenotype was characterized by expression of vimentin and alpha-smooth muscle actin (α-SMA) (11). To incorporate VIC within the hydrogel, ARdH was transferred to a tube, neutralized with NaOH to a final concentration of 10 mM, homogenized and incubated for 15 min on ice. The mixture was then buffered with 10x PBS to a final 1x concentration, followed by centrifugation at 3,000 g for 15 min at 4°C to remove air bubbles. After centrifugation, trypsinized VIC were resuspended in a small volume of cell culture media and incorporated into the gel (10^6^ cells/100 μl cell culture media) to a final density of 5 × 10^5^ VIC/ml ARdH. The mixture was incubated at 37°C and 5% CO_2_ for 2 h to allow collagen assembly and gelling. After incubation, DMEM 5 mM glucose with 10% fetal bovine serum (FBS) was added to the obtained 3D constructs. After 24 h of culture, VEC were seeded (5 × 10^4^/cm^2^) on the surface of VIC encapsulated constructs and incubated as a VEC/VIC co-culture in M200 media supplemented with Low Serum Growth Supplement (LSGS), 10% serum and 1% antibiotics until VEC reached confluence (~48 h). At that point, the co-culture constructs were exposed for different time intervals to normal M200 media supplemented with 5 mM glucose (NG) and media supplemented with 25 mM glucose (HG) (G5146, Sigma).

#### Biocompatibility Assay

VEC and VIC were cultured in the ARdH 3D construct for up to 11 days to determine biocompatibility and cell proliferation. For VEC seeded on the surface, MTT assay (Trevigen, 4890-25-K) was performed, while for encapsulated VIC, total LDH activity (ThermoFisher, 88954) was quantified using the manufacturers' protocol. Results were expressed as mean absorbance values for each group (*n* = 4).

#### Histology and Immunofluorescence

Aortic root tissue and ARdH 3D constructs (*n* = 3) were washed with PBS and fixed in 4% PFA for 1 h at RT. Cryoprotection was performed by successive incubation steps in PBS and 5, 10, 20, and 50% glycerol solutions. After 6 washes in 3% sucrose in 0.1 M phosphate buffer, samples were immersed in OCT compound for 1 h (Neg-50, Thermo Scientific) and cryosections of 7 μm were cut on a Leica Cryotome. Sections were stained with Hematoxylin and Eosin (Vector, H-3502), Picro Sirius Red (Abcam, ab150681) and a modified Russell-Movat pentachrome stain (Nordic BioSite, KSC-L53PIA-1) according to the manufacturer's protocol.

For immunofluorescence, cryosections were brought to room temperature for 10 min and then washed with PBS and blocked in TBS Blotto A (SC-2333, Santa Cruz) with 3% BSA, 0.3% Triton X-100 and 1% cold fish gelatin (Sigma, G7041) for 1 h at room temperature, followed by incubation overnight at 4°C with primary anti-human antibodies: CD31 at 1:100 dilution (SC-376764, Santa Cruz), α-SMA at 1:100 dilution (SC-32251, Santa Cruz), VE-Cadherin at 1:100 (14-1449-82, ThermoFisher), vimentin at 1:500 (SC-6260, Santa Cruz) and endothelin-1 at 1:250 dilution (PA3-067, ThermoFisher). The next day, samples were washed and incubated with Alexa Fluor 594 or FITC conjugated secondary antibodies at a dilution of 1:1,000 for 30′ at RT in the dark. Nuclei were stained with DAPI 10 μg/mL (Sigma, D9542) and samples were mounted in Fluoromount-G (Thermo Scientific, Waltham, MA, USA). Images were acquired using a confocal microscope LSM Airyscan (Carl Zeiss AG, Oberkochen, Germany) and a fluorescence microscope (Olympus IX81, Shinjuku City, Tokyo, Japan) equipped with an XM10 camera. Images were processed using ImageJ software. Mean fluorescence intensity (MFI) of images was calculated for each fluorophore and normalized to the number of cell nuclei stained with DAPI (9 images per sample).

### Transmission Electron Microscopy

For transmission electron microscopy (TEM), samples were fixed with 2.5% glutaraldehyde in 0.1 M cacodylate buffer at room temperature, cut in 1 mm^3^ pieces, and post-fixed in buffered 1% OsO4 with 1.5% K4Fe(CN)6 (potassium ferrocyanide reduced osmium) with pH 7.4 at 4°C for 1 h. Fixed samples were dehydrated in graded ethanol series and embedded in epoxy resin (Agar100 resin, Agar Scientific Ltd. UK). The ultra-thin sections were cut with a diamond knife (Diatome, Switzerland), at 60 nm thickness using an EM UC7 Leica ultramicrotome (Leica Microsystems, Germany) and double-stained with 1% uranyl acetate and Reynold's lead citrate. TEM was performed using a Morgagni 268 transmission electron microscope (FEI Company, Eindhoven, The Netherlands) at 80 kV. Digital electron micrographs were recorded with a MegaView III CD and iTEM-SIS software (Olympus, Soft Imaging System GmbH, Germany).

### Quantitative Real-Time PCR

Total RNA was isolated from VIC and VEC using PureLink RNA Kit (Thermo Fisher Scientific, Waltham, MA, USA) according to the manufacturer's protocol. One microgram of total RNA was used for first-strand cDNA synthesis using MMLV reverse transcriptase (Invitrogen, Carlsbad, CA, USA). Amplification of cDNA and quantification of gene expression was performed using SYBR™ Green I technology and specific primers (Table 1) on a LightCycler 480 System (Roche, Basel, Switzerland). Relative quantification was performed using the comparative C_T_ method with β-actin as reference gene and results were expressed as fold change relative to controls (normal glucose conditions).

**Table 1:** PCR primers sequences used for gene expression evaluation.

**Gene**	**GenBank^®^** **accession number**	**Sequences of oligonucleotide primers**	**Predicted size (bp)**
ET-1	NM_001955.5	Fw: 5′- ctttgagggacctgaagctg-3′ Rv: 5′- agttcttttcctgcttggca-3′	465
VCAM-1	NM_080682	Fw: 5′- gggcacagaatccatttcat-3′ Rv: 5′- tccgtctcattgacttgcag-3′	89
ICAM-1	NM_000201.3	Fw: 5′-ttgggcatagagaccccgtt-3′ Rv: 5′-gcacattgctcagttcatacacc-3′	82
ET_A_	NM_001957	Fw: 5′- tcgggttctatttctgtatgccc-3′ Rv: 5′- tgtttttgccacttctcgacg-3′	143
ET_B_	NM_000115.5	Fw: 5′- gcaaaccgcagagataatgacg-3′ Rv: 5′- ggacacaaccgtgttgatgtatt-3′	205
Vimentin	NM_003380.5	Fw: 5′- agtccactgagtaccggagac-3′ Rv: 5′-catttcacgcatctggcgttc-3′	98
CD31 (PECAM-1)	NM_000442.5	Fw: 5′-ccaaggtgggatcgtgagg-3′ Rv: 5′-tcggaaggataaaacgcggtc-3′	187
VE-cadherin	NM_001795.5	Fw: 5′- ttggaaccagatgcacattgat-3′ Rv: 5′- tcttgcgactcacgcttgac-3′	86
α-SMA	NM_001141945.2	Fw: 5′-actgccttggtgtgtgacaa-3′ Rv: 5′-caccatcaccccctgatgtc-3′	120
β-actin	NM_001101.4	Fw: 5′-catgtacgttgctatccaggc-3′ Rv: 5′-ctccttaatgtcacgcacgat-3′	250

### ELISA Assay

IL-1β levels were measured in conditioned media collected on day 7 from 3D constructs. Media was centrifuged at 3,000 rpm for 10 min and cell debris was discarded. Supernatant aliquots were stored at −80°C until further use. ELISA assay (DuoSet and Quantikine ELISA Kits, R&D Systems) was performed according to the manufacturer's protocol.

### Monocyte Adhesion Under Shear Flow Conditions

Laminar flow assay was performed as previously described (17). Briefly, ARdH was poured on a Ø 35 mm coverslip and VEC were cultured on top. The coverslips were exposed to NG or HG conditions for 7 days and then were assembled in a parallel wall flow chamber (FCS2 Chamber, Bioptechs) and mounted on the stage of an Olympus IX-81 inverted microscope with 4 × and 10 × phase contrast objectives. THP-1 cells (TIB-202, ATCC) were fluorescently labeled using Calcein-AM (100 mM) and suspended (0.5 × 10^6^/ml) in Hanks' buffered salt solution containing 10 mM HEPES, pH 7.4 and 0.5% human serum albumin. Cells were perfused for 15 min into the flow chamber at a rate of 1.5 dyn/cm^2^ while being kept on a 37°C heating block. Mg^2+^ (1 mM) and Ca^2+^ (1 mM) were added to monocytes shortly before the assay. RPMI was perfused for 15 min to wash out non-adherent cells and the remaining firmly adhered cells were quantified by image analysis using Cellsense software (Olympus, Japan) (*n* = 10 images, mean ± SD).

### Statistical Analysis

Statistical analysis was performed with GraphPad Prism 7.0 with data points denoting mean ± standard deviation (SD). Statistical significance is shown as *p*-values obtained via a two-tailed Student's *t*-test when comparing two experimental groups and analysis of variance (One-Way ANOVA) with multiple comparisons when comparing more than two groups. A *p*-value of *p* < 0.05 was considered statistically significant.

## Results

### Characterization of the ARdH 3D Model

In order to obtain a homogeneous hydrogel, complete removal of cells from the tissue matrix is mandatory. Therefore, we extracted and purified genomic DNA after decellularization and evaluated the residual DNA by UV spectrophotometry (NanoDrop). Fresh tissue contained 137.2 ng DNA/mg tissue while decellularized matrix only 0.446 ng DNA/mg. The results showed a 99% reduction in DNA content (<0.5 ng/mg wet tissue) of ARdH compared with AR tissue.

**Figure 1:**
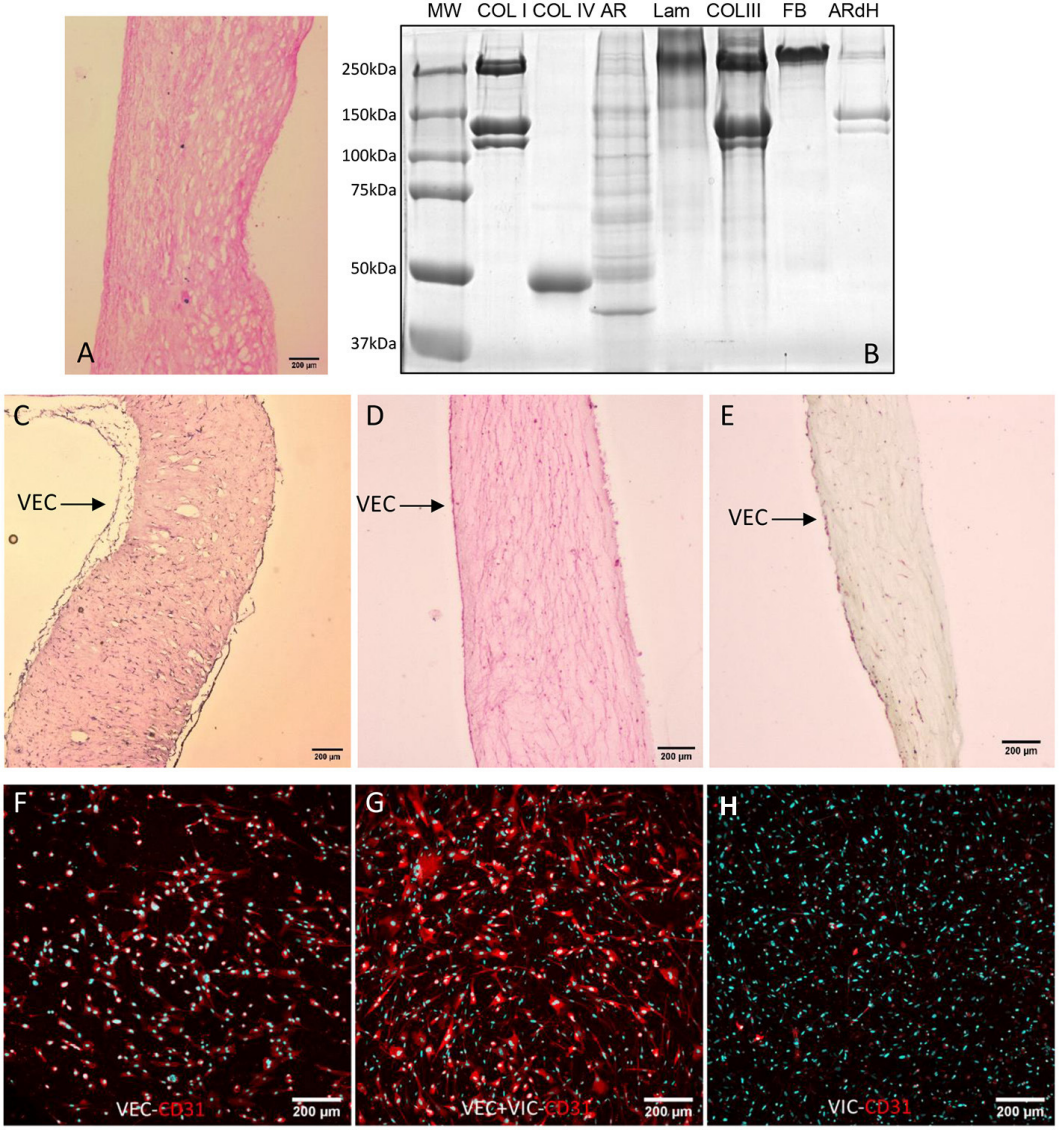
Characterization of ARdH 3D model. **(A)** Histological evaluation by hematoxylin-eosin staining of decellularized aortic root tissue (10X magnification). **(B)** Coomassie staining of 8% Laemmli SDS-PAGE. Lane 1 – molecular weight marker; lane 2 – collagen I (Coll I); lane 3 – collagen IV (Coll IV); lane 4 – porcine aortic root before decellularization (AR); lane 5 – laminin (Lam); lane 6 – collagen type III (Coll III); lane7 – fibronectin (FBn); lane 8 – ARdH. Cross-sections of ARdH populated with valvular cells and stained with **(C)** Hematoxylin-eosin, **(D)** Picrosirius red and **(E)** with Movat Pentachrome. CD31 expression in VECs (surface of ARdH) **(F)**, VECs/VIC (Z-stack reconstruction) **(G)** and VICs (inside ARdH); **(H)** Immunofluorescence staining of CD31 (red) and nuclei DAPI-cyan, after 24 h of culture. Z-stack confocal reconstruction.

We further analyzed sections of decellularized tissue by histology (Figure 1A) and evaluated by SDS-PAGE the protein composition of ARdH compared with AR tissue and specific matrix recombinant proteins. The gel electrophoretic pattern observed in ARdH hydrogel is very similar to that of collagen I, suggesting that collagen type I is a major constituent (Figure 1B).

Histological evaluation of ARdH populated with valvular cells revealed a continuous monolayer of VEC on the hydrogel top surface and VIC uniformly distributed inside the hydrogel (Figures 1C–E). A fibrillar appearance of ARdH was seen by Picrosirius red staining (Figure 1D). To further distinguish the endothelial cells from the 3D construct, we performed immunofluorescence using CD31, a specific marker for endothelial cells. The fluorescence microscopy pictures revealed that only the cells from the surface of 3D construct were positive for CD31 (Figures 1F,G), whereas in the core of the construct CD31 was not present (Figure 1H).

**Figure 2:**
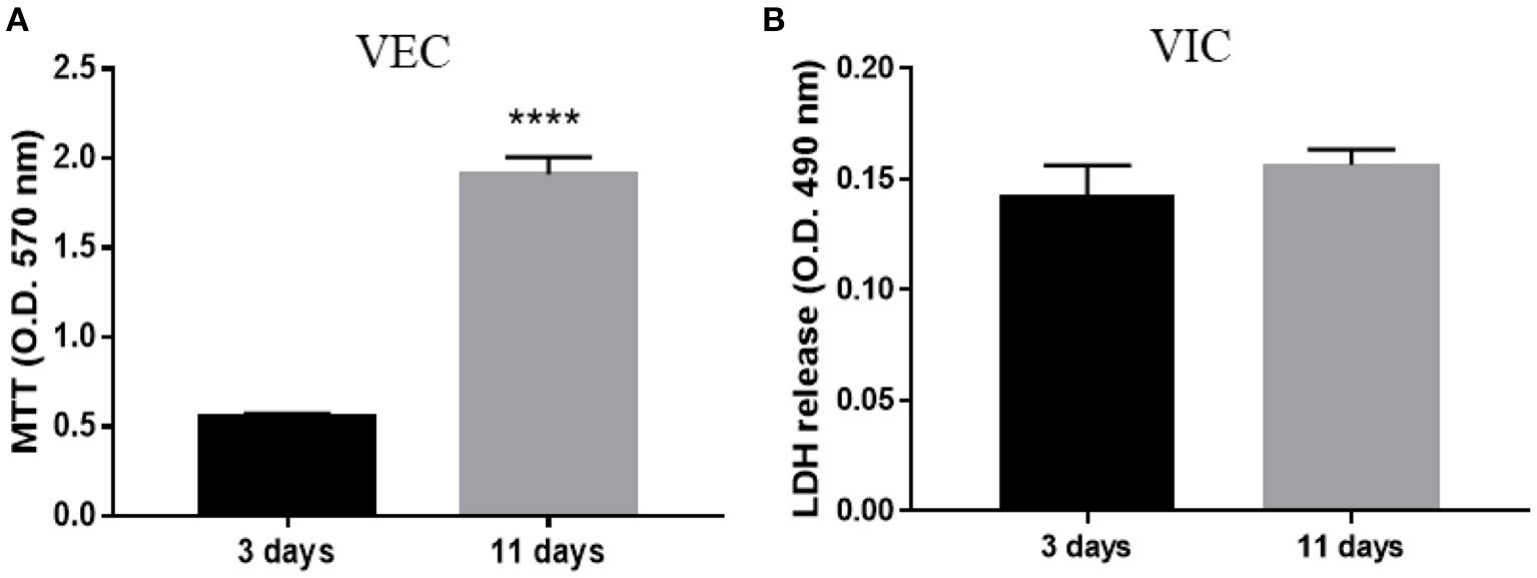
Biocompatibility of ARdH with valvular cells. **(A)** Viability of VEC after 3 and 11 days of culture. The number of VEC was significantly increased after 11 days compared with 3 days of culture. **(B)** Total LDH measurements: the graph shows the level of LDH released by VIC encapsulated in the ARdH construct. There are no changes in the LDH levels at 3 days compared with 11 days (^****^p < 0.0001, *n* = 4).

The viability of valvular cells from the 3D constructs was investigated using live/dead staining (Hoechst/PI) after 24 h (Supplementary Figure 1), and MTT/LDH after 72 h and 11 days of culture. Approximately 95% of cells from the 3D model remained viable after 24 h of culture (Supplementary Figure 1). After 3 and 11 days, the number of VEC exceeded the number of cells initially encapsulated per construct, as evaluated by MTT assay (Figure 2A), while VIC did not exhibit cytotoxicity over time (Figure 2B). These results suggest that cells from constructs were viable and VEC proliferate.

### HG Increases Intermediate Filaments Abundance and Attenuates Intercellular Junctions in VEC

**Figure 3:**
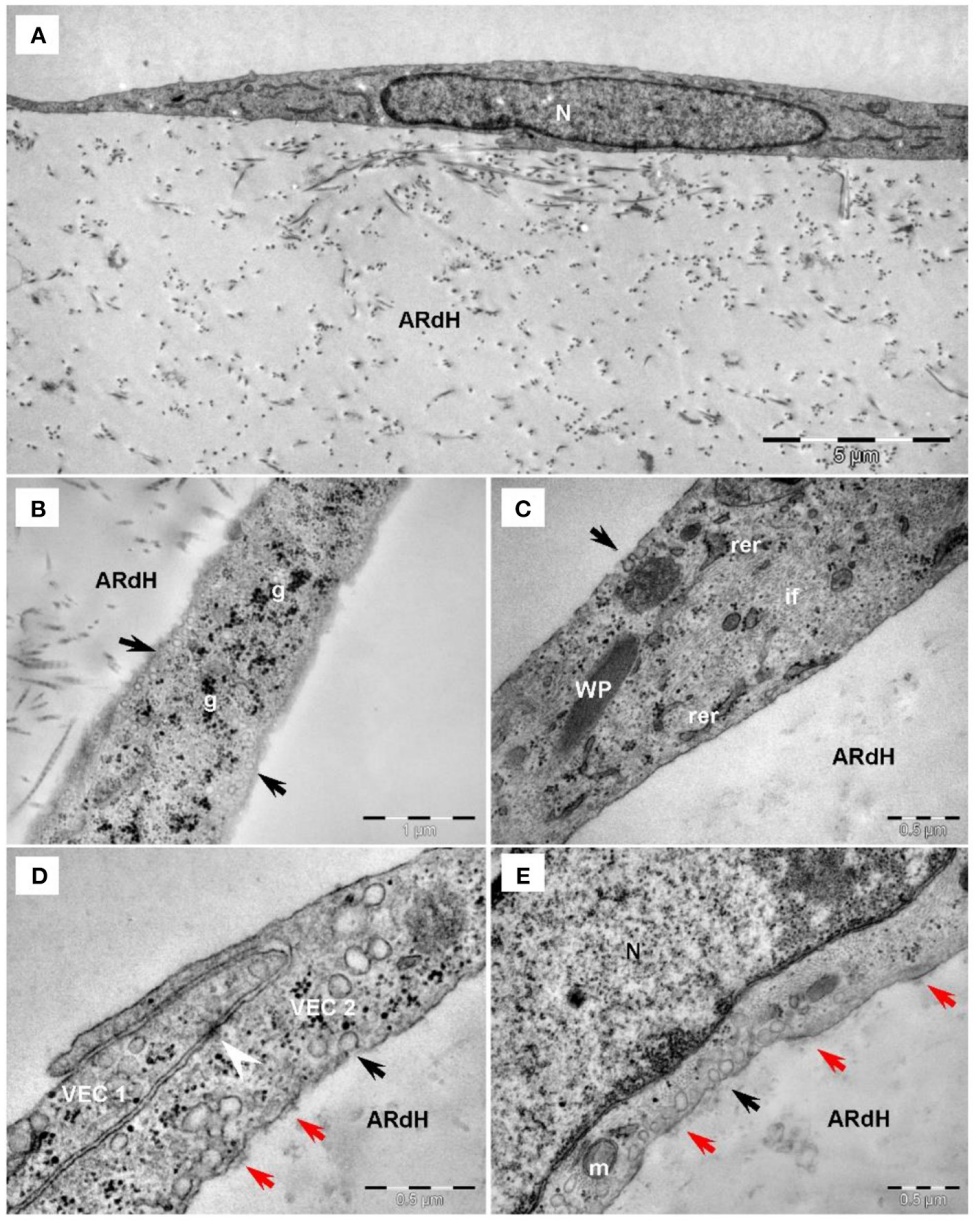
Transmission electron microscopy of VEC exposed to normal glucose conditions on the hydrogel construct (ARdH). **(A)** VEC show a central euchromatic nucleus (N) and long, flat cellular extensions, a few endoplasmic reticulum cisterna and mitochondria. ARdH contains fibrillary collagen. **(B)** Numerous caveolae are visible on the cellular membrane and in the cortical space (black arrows). Glycogen particles (g) are spread throughout the cytoplasm. **(C)** Intermediate filaments (if) are well-represented. Weibel-Palade bodies (WP) are present in VEC. Rough endoplasmic reticulum (rer) showed few cisternae. **(D)** Intercellular connections between VEC1 and VEC2, recessus adhaerens type (or plug and socket), are fastened by adhaerens junction (arrowhead). Focal adhesion points (red arrows) connect VEC to the support construct. **(E)** Focal adhesion points (red arrows) connect the VEC to the hydrogel construct (AR). Black arrows indicate caveolae. m, mitochondrion; N, nucleus.

The ultrastructural features of VEC from the 3D construct exposed to HG conditions for 48 h or 7 days and compared to NG were evaluated by TEM imaging. In NG, long and slender VEC were observed to cover the construct (Figure 3A). Cells presented a large central, euchromatic nucleus and organelles in various amounts: mitochondria, rough endoplasmic reticulum (RER) and free ribosomes, Golgi system, lysosomes, glycogen β particles and intermediate filaments. Caveolae (Figure 3B) and Weibel-Palade bodies were present in VEC (Figure 3C). VEC displayed a flat morphology and presented clear intercellular junctions between overlapping prolongations (Figure 3D). VEC are connected with extracellular matrix resembling basal lamina by focal contacts (Figure 3E).

**Figure 4:**
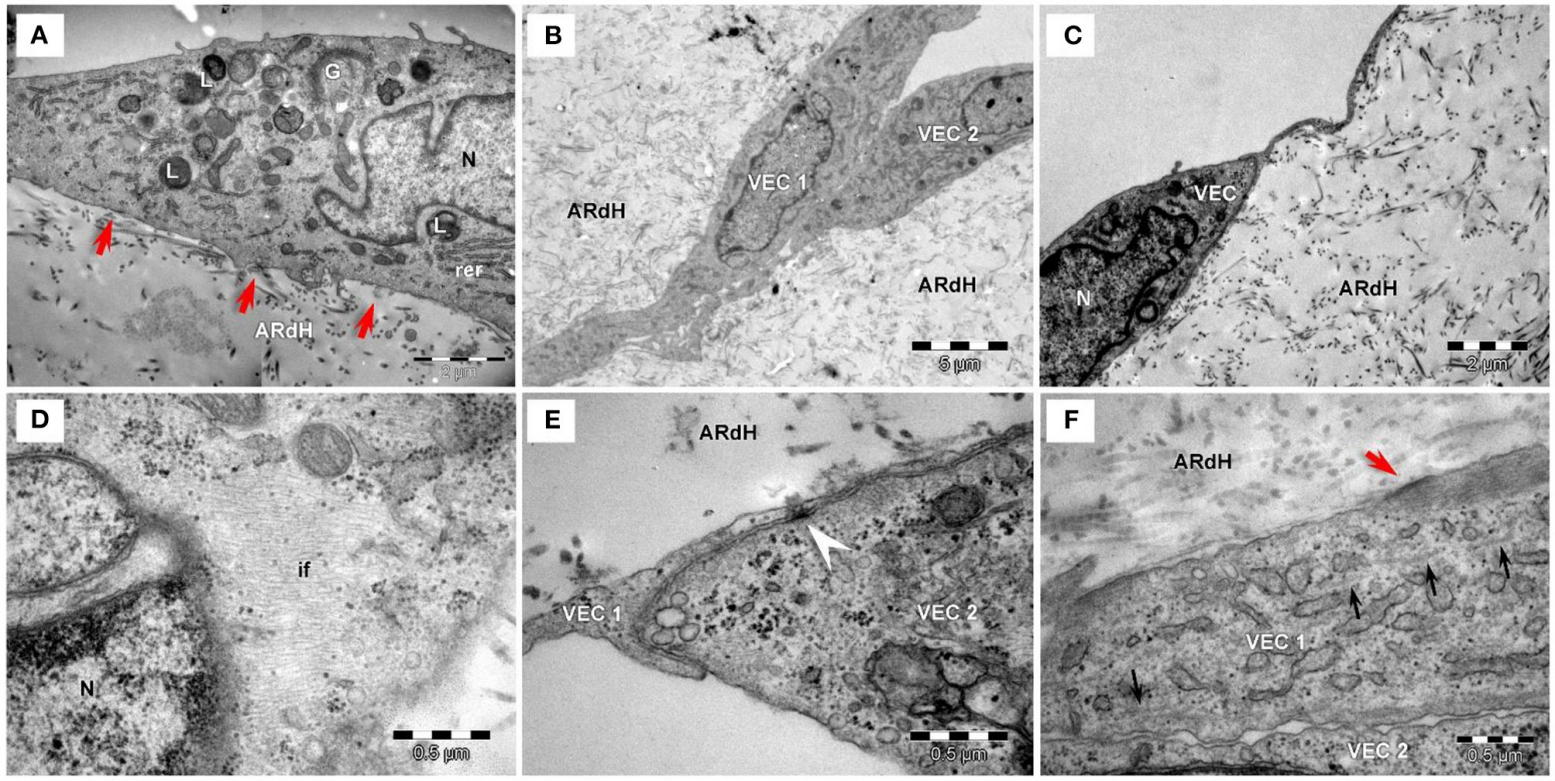
Transmission electron microscopy of VEC exposed to high glucose conditions for 48 h **(A)** or 7 days **(B–F)** on the hydrogel construct. **(A)** VEC in hydrogel (ARdH) show endothelial protrusions, a developed prominent Golgi system (G), rough endoplasmic reticulum (rer), numerous lysosomes (L). Caveolae are visible on the plasma membrane. Focal adhesion junctions (red arrows) connect VEC to the hydrogel. **(B)** VEC (1, 2) present extended bodies in the hydrogel and with more numerous organelles and prominent cytoplasm. **(C)** VEC with a smaller body and heterochromatic nucleus. **(D)** Abundant intermediate filaments (if) are distributed throughout the cytoplasm. **(E)** Adherens junctions (arrowhead) connect two different VEC (1, 2). **(F)** Intercellular junctions between VEC (1, 2) are less developed. Focal adhesions (red arrows) connect VEC with ARdH hydrogel. Microtubules (small black arrows) are visible into the cytoplasm.

Compared to NG conditions, after 48 h of exposure to HG conditions, VEC displayed endothelial protrusions, a more developed cytoskeleton, numerous caveolae visible lysosomes and focal adhesion junctions connecting VEC to the hydrogel (Figure 4A).

After 7 days of HG exposure, VEC extended bodies in the hydrogel (Figure 4B), exhibited smaller bodies, a heterochromatic nucleus (Figure 4C), numerous mitochondria and lysosomes (Figure 4A), abundant intermediate filaments (Figure 4D) and microtubules, as well as areas of focal adhesion junctions (Figure 4E). Moreover, compared to NG conditions, VEC in HG showed attenuated intercellular and cell-to-matrix junctions (Figure 4F).

### Increased Glucose Concentration Induces EndMT in VEC From the 3D Model

The ultrastructural modifications revealed by TEM analysis show an increased abundance of intermediate filaments and attenuation of intercellular junctions induced by HG, features known to be associated with a mesenchymal phenotype of VEC and therefore a possible initiation of the EndMT process. Previously, EndMT was detected in calcific aortic valve disease (18), and HG was shown to induce EndMT in other types of EC (19, 20). Therefore, in this study, we followed the changes induced by HG on endothelial and mesenchymal cell markers in VEC from the 3D construct exposed to HG.

**Figure 5:**
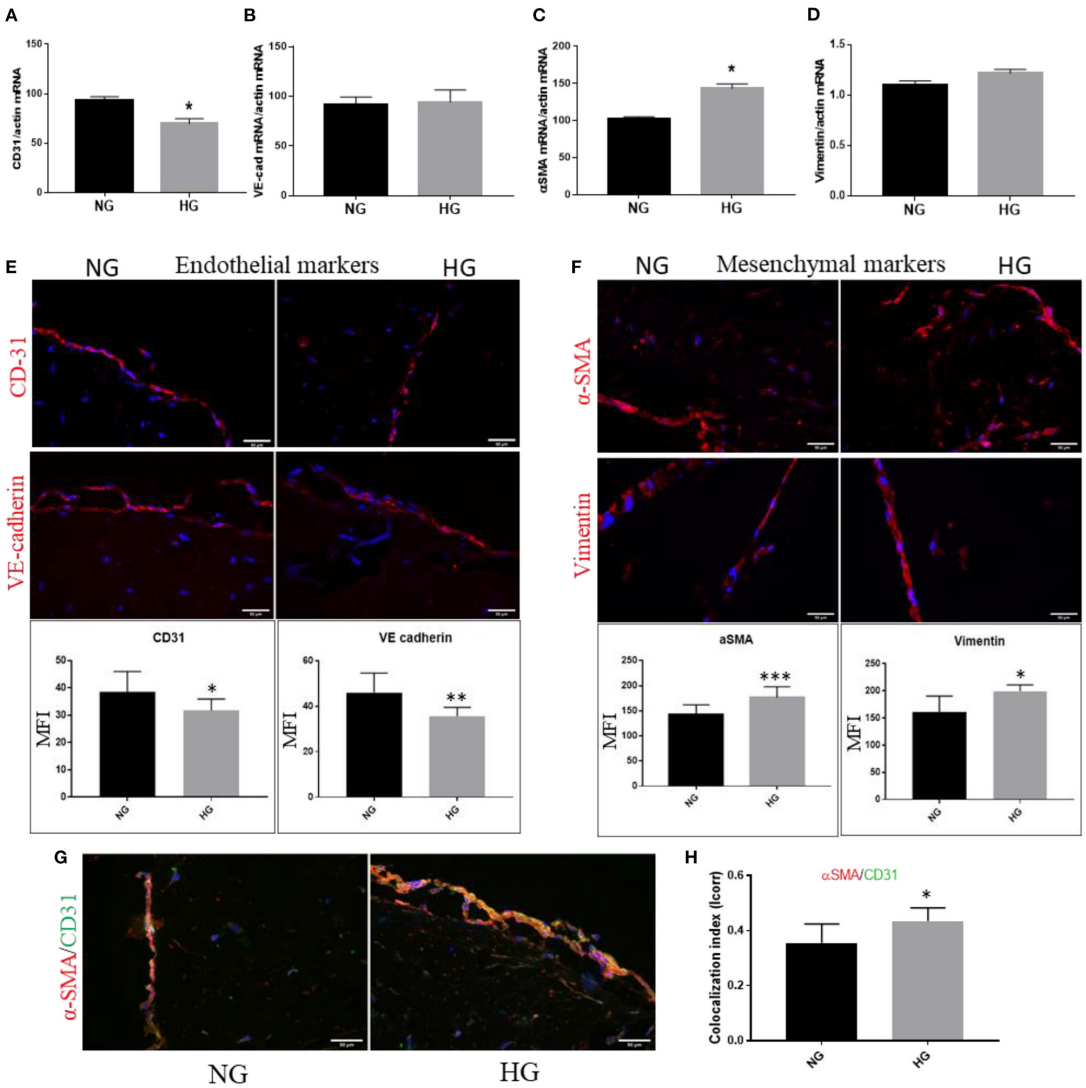
High glucose induces endothelial–mesenchymal transition in VEC from 3D construct. mRNA expression of endothelial markers CD-31 and VE-cadherin **(A,B)** and mesenchymal markers α-SMA and vimentin **(C,D)** VEC isolated from 3D-constructs cultured for 7days in NG or HG conditions. *n* = 3, ^*^p < 0.05, ^**^p < 0.01, ^***^p < 0.001. **(E,F)**. Immunofluorescence staining of endothelial markers – CD31 and VE-cadherin **(E)**, of mesenchymal markers – αSMA and vimentin **(F)** and co-localization of endothelial CD31 (green) with mesenchymal marker αSMA (red) in valvular cells after 7 days of normal (control) and high glucose exposure **(G)** - mean fluorescence intensity (MFI) of images was calculated for the fluorophore and normalized to the number of cell nuclei stained with DAPI (*n* = 3). Colocalization index of αSMA/CD31 in NG vs. HG, as obtained by calculating index of correlation (Icorr) **(H)**.

We evaluated the gene expression of endothelial and mesenchymal markers known to be modified when endothelial cells undergo the transition toward a mesenchymal phenotype: CD31, VE-cadherin, α-SMA and vimentin. The results showed that at 7 days of exposure to HG, VEC exhibited a significantly reduced gene expression of EC marker CD31 and a significantly increased mesenchymal marker α-SMA (Figures 5A,C). Gene expression of VE-cadherin and vimentin was not found significantly modified at this time (Figures 5B,D). To further investigate these markers at the protein level, 3D construct cryosections were immunoassayed for the mentioned markers. The semiquantitative analysis performed on immunofluorescence images revealed that EC markers CD31 and VE-cadherin were decreased on VEC from the 3D construct while the mesenchymal markers α-SMA and vimentin expression were significantly increased (Figures 5E,F).

To further identify the EndMT transformation of valvular endothelial cells, double-staining for CD31 and α-SMA was performed on sections obtained from the 3D construct. At the VEC level, in HG conditions we detected co-localization of the mesenchymal biomarker α-SMA with the endothelial marker CD31 (Figure 5G). Colocalization index of α-SMA/CD31 in NG vs. HG is presented in Figure 5H.

### High Glucose Increases Expression of ET-1 in VEC and Blockage of Endothelin Receptors ET_A_ and ET_B_ Reduced IL-1β and MCP-1 Secretion From 3D-construct Exposed to HG Conditions

**Figure 6:**
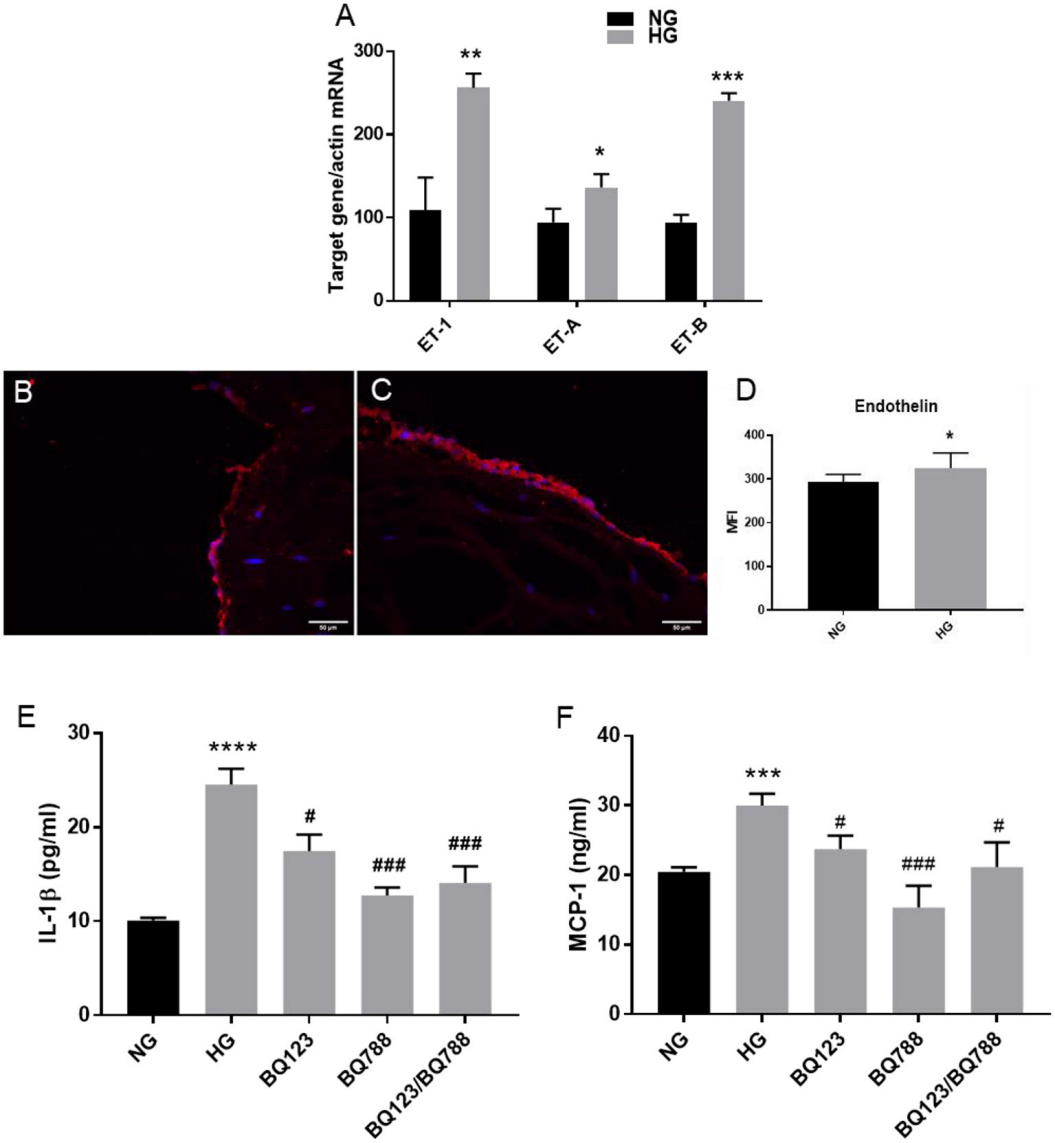
Endothelin-1 expression is increased by HG levels in VEC; inhibition of endothelin-1 receptor A/B with the specific inhibitors BQ123, BQ788 decreased the secretion of inflammatory molecules IL-1β and MCP-1. **(A)** Gene expression of ET-1 and ET-1 receptors (ET-A and ET-B) in VEC isolated from 3D-constructs after 7 days of normal (control) and high glucose exposure, as evaluated by Real-Time PCR. *n* = 3. **(B,C)** Representative images of ET-1 protein expression, as evaluated by immunofluorescence. Cryosections were stained with ET-1 primary antibodies and Alexa coupled secondary antibodies (red staining). Nuclei were stained with DAPI (blue staining). **(D)** Protein expression of ET-1 in valvular cells - mean fluorescence intensity of images was calculated for the fluorophore and normalized to the number of cell nuclei stained with DAPI. **(E,F)** Soluble MCP-1 and IL-1β protein secreted in the conditioned media by VEC from 3D-construct exposed to HG conditions in presence or absence of inhibitors for ET-1 receptors BQ-123 and BQ788 and NG for 7 days, as determined by ELISA assay. *n* = 3, ^*^p < 0.05, ^**^p < 0.01, ^***^p < 0.001, ^****^p < 0.0001, ^#^*p* < 0.0.5, ^*###*^*p* < 0.001. ^*^NG vs. HG, ^**#**^HG vs. HG + inhibitors.

Since there is evidence that aortic stenosis is characterized by distinct upregulation of ET-1 and its target receptor Endothelin Receptor type A (ET_A_), promoting inflammation and fibrosis (21), we further searched if HG affects the endothelin system in VEC from our 3D model. The results showed that HG induced up-regulation of ET-1 mRNA expression levels in VEC (Figure 6A). The protein expression of ET-1 was also found increased in VEC exposed to HG compared to controls, as evaluated by immunofluorescence (Figures 6B–D). In addition, the gene expression of both ET_A_ and Endothelin Receptor type B (ET_B_) were found significantly elevated in HG exposed VEC as compared with NG condition (Figure 6A).

There is evidence that endothelin is an early proinflammatory agent in diabetes, that upregulates the expression of inflammatory markers in endothelial cells, including IL-1β and MCP-1 (22). We have recently shown that chronic HG exposure of a gelatin methacrylate 3D model of human aortic valve increased expression of the inflammatory markers: IL-1β and MCP-1 in VEC (10). To evaluate if the endothelin pathway is involved in IL-1β and MCP-1 secretion, we exposed the 3D construct to HG in the presence of selective endothelin receptor antagonists for the receptor A (BQ-123) or receptor B (BQ788), or both. Soluble protein levels of IL-1β (sIL-1β) and MCP-1 (sMCP-1) released in the conditioned media (Figures 6E,F) were quantified by ELISA. The results showed that exposure of the 3D model to HG in the presence of the inhibitor for ET_A_ (BQ-123) significantly reduced sIL-1β and sMCP-1, while the inhibitor for ET_B_ (BQ788) significantly reduced to control levels sIL-1β and sMCP-1; moreover, the presence of both inhibitors reduced sIL-1β and sMCP-1 to the control levels. These results suggest that endothelin signaling might be involved in the modulation of HG-induced inflammatory molecules.

### High Glucose Induced Cell Adhesion Molecules Are Functional in Monocyte Adhesion to Valvular Endothelial Cells

**Figure 7:**
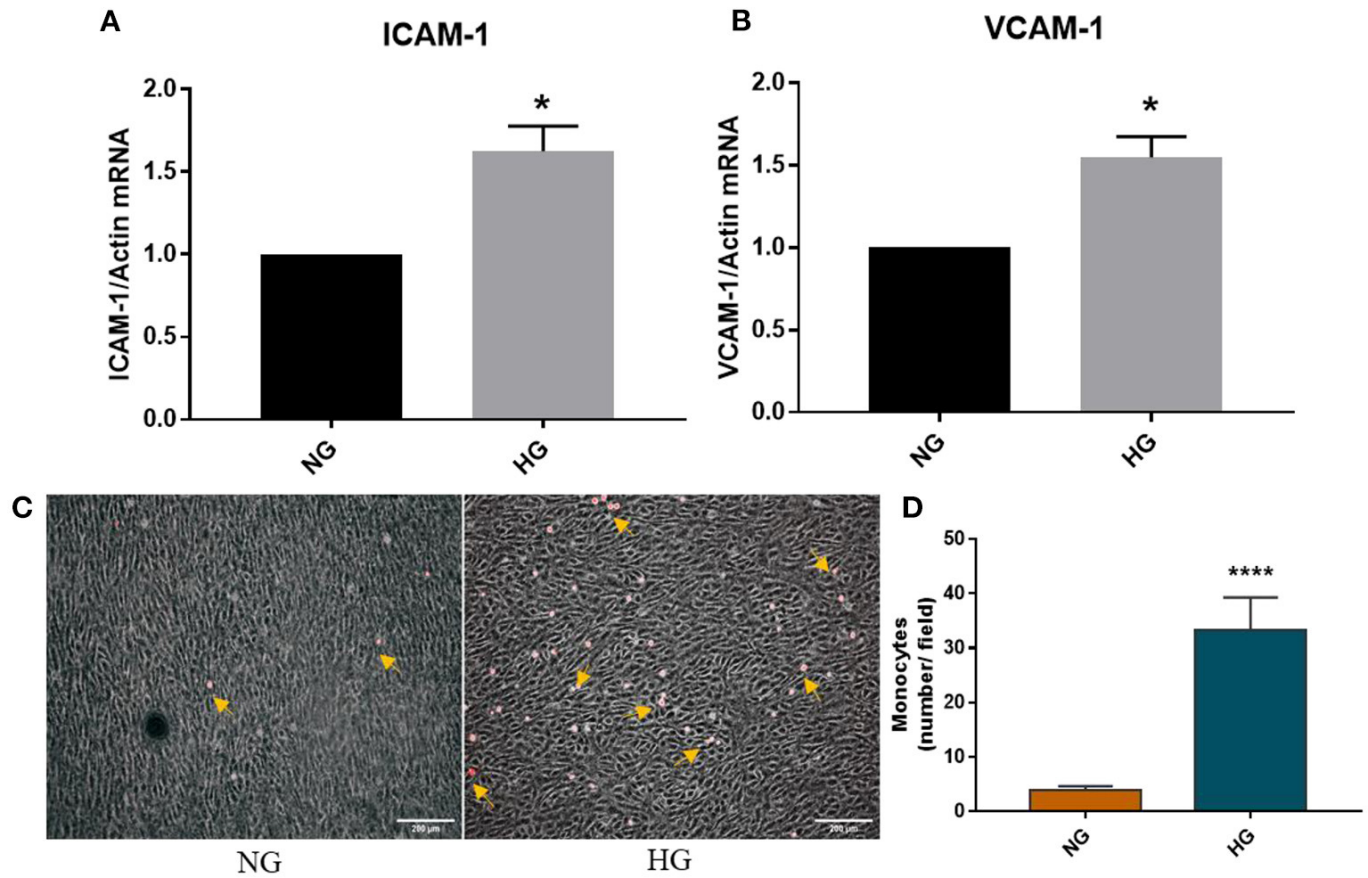
HG induced an adhesive phenotype in VEC in the 3D ARdH model. **(A,B)** Gene expression of ICAM-1 and VCAM-1 in VEC from 3D-constructs exposed to NG or HG for 7 days, as evaluated by Real-Time PCR. *n* = 4, ^*^p < 0.05 NG vs. HG. **(C,D)** Cell adhesion of THP-1 monocytes (yellow arrows) to the endothelial monolayer (phase contrast) on 3D constructs exposed to NG or HG, in laminar flow condition. The number of firmly attached monocytes to HG-exposed 3D constructs was 7 times higher compared with control NG. ^****^p < 0.0001 vs. NG.

Endothelial dysfunction is associated with increased expression of cell adhesion molecules that further mediates increased monocytes adhesion. Previously, using normal 2D culture and bovine VEC, we have demonstrated that high glucose induces enhanced monocyte adhesion to valvular endothelial cells via a mechanism involving ICAM-1, VCAM-1, and CD18 (9). Moreover, we have recently shown that chronic HG increased gene expression of cell adhesion molecules VCAM-1 and E-selectin in a 3D model of human aortic valve based on methacrylated gelatin (10). In the present 3D model based on a native extracellular matrix, we similarly found that HG significantly increased VCAM-1 but also ICAM-1 gene expression after 7 days of HG exposure (Figures 7A,B). To evaluate the functional role of the cell adhesion molecules increased by HG in VEC, we further investigated the adhesive interaction between fluorescently labeled monocytes and endothelial cells by exposing the 3D model to HG in laminar flow conditions. These results showed that monocytes adhered at a significantly higher rate to HG than NG-exposed VEC (Figures 7C,D), demonstrating the increased adhesiveness of VEC in HG conditions.

## Discussions

Valvular endothelial cells have leading roles in maintaining valvular integrity and homeostasis, and dysfunctional VEC are a major contributor to aortic valve disease. There is evidence that VEC switch to a secretory and pro-inflammatory phenotype in early diabetes in animal models (8) and *in vitro* models (9–11). In this work, we extended these studies and further evaluated the impact of HG on the phenotypic switch of VEC in a new 3D model of human aortic valve, based on a native extracellular matrix derived from aortic root.

The main novel findings of our study are that VEC from the 3D model exposed to chronic HG exhibited: (i) attenuated intercellular junctions and an abundance of intermediate filaments; (ii) EndMT activation, as revealed by decreased expression of endothelial markers: CD31 and VE–cadherin and increased expression of the mesenchymal markers: α-SMA and vimentin and coexistence of CD31 and α-SMA in VEC; (iii) increased expression of molecules associated with an inflammatory process: ET-1 and its receptors ET_A_ and ET_B_; (iv) reduced expression of inflammatory biomarkers IL-1β and MCP-1 upon blockage of ET-1 receptors.

The results are particularly relevant as they were obtained on VEC cultured in a physiologically appropriate matrix environment by using ECM derived from porcine aortic root. The ECM was obtained using a slightly modified version of the method described previously by Sierad's group, where it has been shown that the main components of ECM—elastin fibers, collagen and GAGs—remain almost intact following the decellularization of the porcine aortic valve (15). The biochemical analysis of our developed hydrogel showed that collagen I and III were the main components of the matrix, and histological analysis revealed that the structure of the 3D model was similar to the aortic valve structure. Importantly, pathological changes of the aortic valve are developed in fibrosa, which contains mostly type I and III collagen (23), recommending our 3D construct as a suitable model to explore HG impact on VEC phenotype, to better understand valvular cells response to hyperglycemia.

Therefore, ultrastructural investigation of human VEC revealed that HG attenuated intercellular junctions and increased the number of caveolae, intermediate filaments and microtubules. Similar modifications were observed in valvular endothelium at the cytoskeleton level in an animal model of combined hyperlipidemia and hyperglycemia (8). Importantly, cytoskeleton remodeling is a feature associated with an EndMT process (24).

Recently, EndMT has been shown to be involved in the pathogenesis of CAVD and specific stimulants, cellular modifications and pathways potentially involved were debated (18). Our data showed that VEC from 3D constructs exposed to HG exhibited increased mesenchymal markers vimentin and αSMA, decreased expression of endothelial markers VE-cadherin and CD31 and increased colocalization in VEC of CD31 and αSMA, indicating that HG induced EndMT transition of VEC. This is the first report, to our knowledge, demonstrating that HG induced EndMT in VEC in a 3D model of the human aortic valve. EndMT triggered by diabetic conditions might accelerate osteogenic and calcific programs in VEC, but future investigations need to clarify these matters.

Accumulating evidence suggests that EndMT represents a key link in the complex interactions between inflammatory stress and endothelial dysfunction (24). Since dysfunctional EC were previously associated with increased levels of ET-1 (25) and ET-1 was found as a proinflammatory agent in early diabetes (14), we explored if HG activates the endothelin system in valve pathology. Using our 3D model exposed to normal or high glucose concentrations, we found that ET-1 and its receptors (ET_A_ and ET_B_) were increased by HG in VEC.

The importance of ET-1 in diabetes came from studies showing that HG increased ET-1 expression in human aortic EC and EC lines (13) and that dysregulation of the ET-1 system plays a major role in the onset and progression of micro-and macrovascular disorders associated with diabetes mellitus (26). There is also evidence that ET-1 and its receptors are increased in aortic stenosis and it was suggested that many features which are typical to human calcified aortic valve including atherosclerosis lesion formation and calcification, may be consequences of increased paracrine/endocrine production of ET-1 in aortic valve (21).

In addition, we showed that specific antagonists for the endothelin receptors ET_A_ and ET_B_ had an inhibitory effect on IL-1β and MCP-1 secretion induced by HG, suggesting further, the possible role of ET-1 in EndMT transition and revealing the endothelin system as a therapeutic target for valve disorders. Moreover, in the present ARdH model of human aortic valve, similarly as in our previously described 3D model based on methacrylated gelatin (11), the inflammatory molecule IL-1β was up-regulated by HG. Since IL-1β is known to be involved in phenotypic transitions of endothelial cells - mainly in EndMT, we might suppose that IL-1β is involved in EndMT transition of VEC. We may speculate that ET-1 and inflammatory IL-1β induced by HG in our model might contribute to EndMT activation in VEC by HG, but future experiments need to be done to clarify this matter. Previously, both ET-1 and IL-1β were found involved in inducing EndMT transition of endothelial cells from other vascular beds (27, 28). *In vivo*, the role of ET-1 in EndMT was first examined in experimentally induced diabetes mellitus in ET-1 knockout mice and it was shown that ET-1 promoted the development of cardiac fibrosis (24). Additional studies showed that silencing of the ET-1 gene abrogated the phenotypic transition of human endothelial cells to a fibroblast phenotype and that this effect occurred through the inhibition of TGF-β signaling (27). IL-1β induced EndMT in human dermal microvascular EC by increasing the expression of mesenchymal markers such as α-SMA, type I collagen, and calponin and inhibiting the expression of vWF (28). Another study reported that a combination of cytokines, including TNF-α, IL-1β, and TGFβ, is more powerful than a single cytokine for inducing EndMT (12).

Based on all this data, we may suppose that HG, by inducing increased expression of ET-1 and inflammatory molecules, promotes VEC transition to EndMT and increases VEC adhesive function, processes that might contribute to the progression of CAVD in diabetic conditions.

In conclusion, our current data indicate that chronic HG induces EndMT in VEC and indicates endothelin as a possible target to reduced HG-induced increased inflammation in VEC.

## Data Availability Statement

The original contributions presented in the study are included in the article/Supplementary Material, further inquiries can be directed to the corresponding author/s.

## Ethics Statement

The studies involving human participants were reviewed and approved by the Ethics Committee of the Institute of Cellular Biology and Pathology Nicolae Simionescu. Primary VEC and VIC were harvested from non-calcified cusps (or portions of the cusp) of human calcified aortic valves obtained from CAVD patients who underwent surgical valve replacement according to Dr. Carol Davila Central Military Emergency University Hospital protocol. The investigation was carried out according to the principles outlined in the Declaration of Helsinki for experiments involving human samples. The participants gave their written informed consent by signing the appropriate paperwork and respecting their anonymity and privacy rights. The animal study was reviewed and approved by the Ethics Committee of the Institute of Cellular Biology and Pathology Nicolae Simionescu. Fresh porcine hearts were obtained from a local abattoir and the aortic roots were freshly excised.

## Author Contributions

IM and EB conceptualization, design of the study, and writing—original draft preparation. SC methodology and performed the experiments. LC, RM, MV, MT, AM, and M-LV performed the experiments and analyzed the data. ID provided the human samples and methodology. MG performed TEM experiments. AS and DS methodology and revision of the manuscript. All authors contributed to the article and approved the submitted version.

## Conflict of Interest

The authors declare that the research was conducted in the absence of any commercial or financial relationships that could be construed as a potential conflict of interest.

## Publisher's Note

All claims expressed in this article are solely those of the authors and do not necessarily represent those of their affiliated organizations, or those of the publisher, the editors and the reviewers. Any product that may be evaluated in this article, or claim that may be made by its manufacturer, is not guaranteed or endorsed by the publisher.
