# Supplementary material for: High Glucose Induced Changes in Human VEC Phenotype in a 3D Hydrogel Derived From Cell-Free Native Aortic Root
Source: Front Cardiovasc Med. 2021 Aug 12;8:714573. doi: 10.3389/fcvm.2021.714573 (PMC8387830; doi:10.3389/fcvm.2021.714573)
Supplement: Supplementary file 1 [file Data_Sheet_1.DOCX]

***Supplementary Figure 1.*** *Live/dead assay for VIC (top row) and VEC (bottom row) after 24h of cultivation in ARdH; yellow arrows indicate dead cells.*


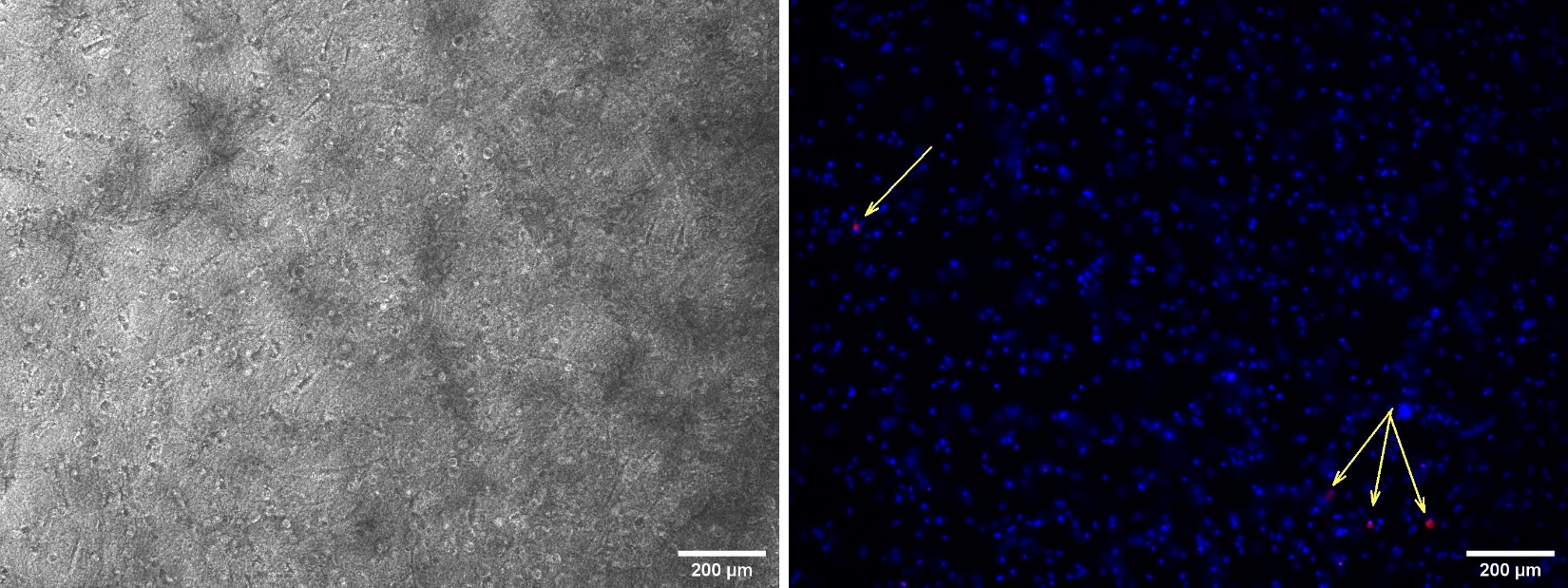

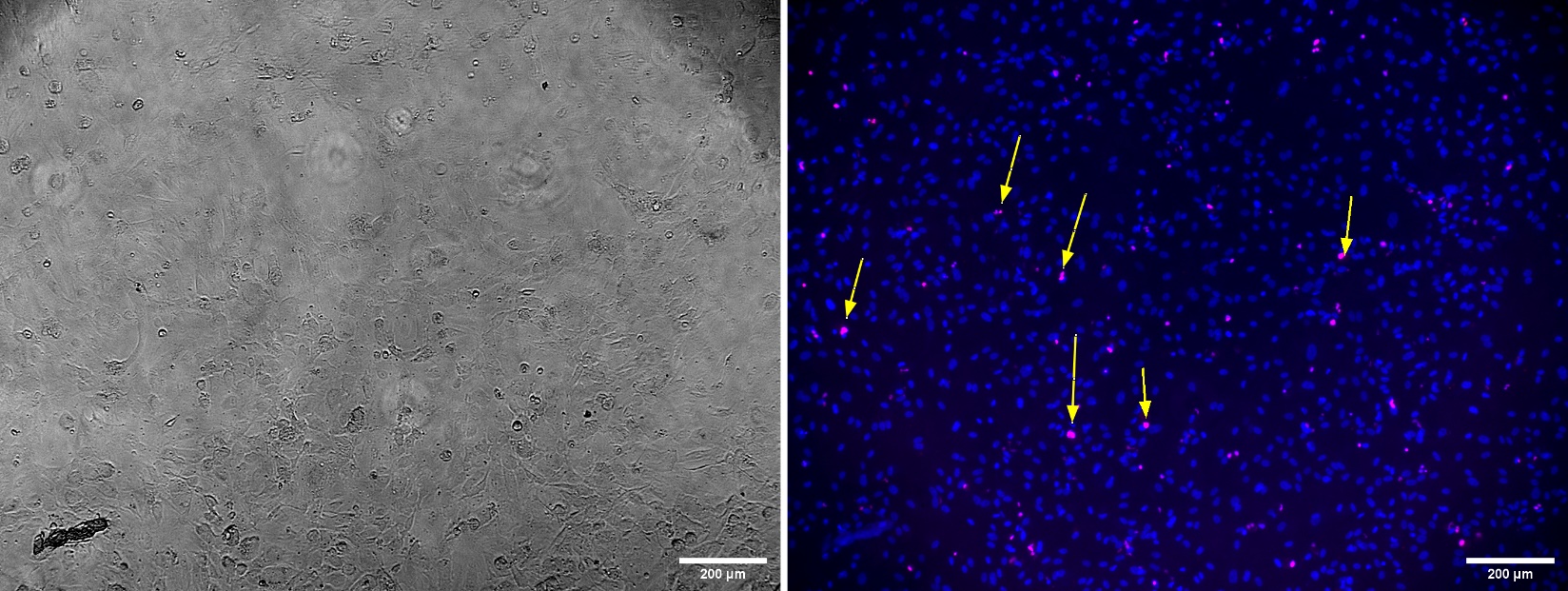


PH Hoechst/PI

VIC

VEC
